# Editorial—The Lifelong Continuum of Pediatric Cardiovascular Care: New Insights from a Growing Field

**DOI:** 10.3390/children13010101

**Published:** 2026-01-10

**Authors:** Sara Moscatelli, Giorgia Rocchetti, Massimo Mapelli

**Affiliations:** 1 Centre for Inherited Cardiovascular Diseases, Great Ormond Street Hospital, London WC1N 3JH, UK; sara.moscatelli90@gmail.com; 2 Institute of Cardiovascular Sciences, University College London, London WC1E 6BT, UK; 3 Paediatric Cardiology Unit, Royal Brompton Hospital, London SW3 6NP, UK; 4 Centro Cardiologico Monzino, IRCCS, 20138 Milan, Italy; giorgia.rocchetti@unimi.it; 5 Department of Clinical Sciences and Community Health, Cardiovascular Section, University of Milan, 20122 Milan, Italy

## 1. Introduction

Pediatric cardiology remains a fascinating yet challenging niche within medicine. Although the number of affected patients is small compared with the adult population, their conditions are often far more complex. For most of the general public, congenital or inherited heart disease remains an abstract concept—something that is only truly understood when experienced within a family. Even within the broader medical community, there is still a tendency to underestimate how profoundly pediatric diseases differ from adult ones—not only in their etiology but also in their natural history, management, and long-term implications [1,2,3].

The pediatric population itself is far from homogeneous. It encompasses a wide range of developmental stages—from the fragile physiology of the newborn to the dynamic cardiovascular adaptations of adolescence. Each phase brings unique diagnostic and therapeutic challenges, making it even harder to generalize findings or establish uniform treatment pathways [2,3].

Conducting research in this field is uniquely demanding. Rare diseases, by definition, involve small patient populations, making it difficult to achieve the sample sizes required for statistical power or multicenter collaboration. Congenital heart diseases (CHD), in particular, encompass a wide range of anatomical and functional presentations, which often defy simple classification and comparison. Moreover, differences in local expertise and institutional practices further complicate the design of multicentric studies and the harmonization of protocols.

The ethical dimension adds another layer of complexity. In pediatric research, every new treatment or procedure must be balanced against the imperative to protect vulnerable patients [4]. Clinical trials are often limited, and pediatric cardiology frequently depends on the adaptation of advances first developed for adult patients. New drugs, interventional techniques, and technologies—while promising—are often used off-label or introduced cautiously after extensive adult experience [1,4].

**Figure 1:**
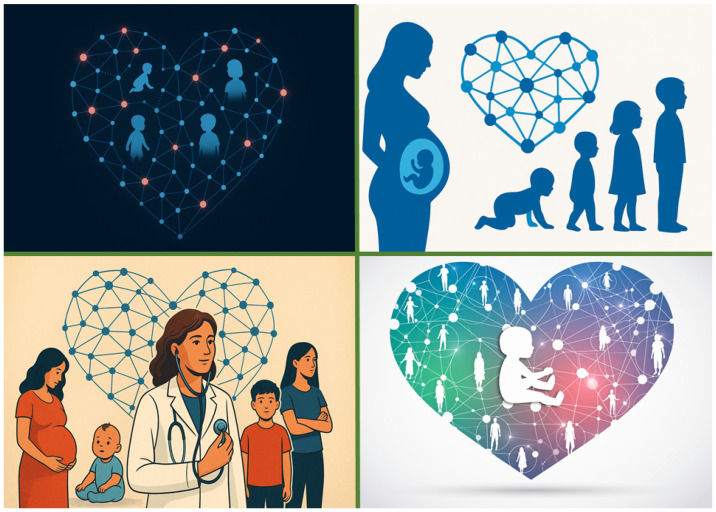
**Conceptual illustrations generated with AI (ChatGPT-4-Turbo (2024) and Adobe Firefly version 2 (2023)).** The images depict interconnected nodes forming heart shapes, symbolizing the collaborative and data-driven nature of modern pediatric cardiology. The silhouettes of children at different developmental stages represent the diversity and complexity of the pediatric population, from infancy to adolescence.

For all these reasons, collaboration and data sharing are key to progress. Pooling expertise and harmonizing datasets can help overcome the limitations imposed by small numbers and fragmented experiences. In this context, artificial intelligence (AI) and machine learning are emerging as powerful allies—tools capable of integrating data from diverse centers, recognizing patterns invisible to the human eye, and potentially redefining diagnostic and prognostic paradigms for rare pediatric diseases [5] (Figure 1).

This Special Issue (*Research Progress of the Pediatric Cardiology: 3rd Edition*) gathers a range of studies reflecting the diversity, creativity, and collaborative spirit that define contemporary pediatric cardiology. From imaging innovations and predictive modeling to simulation and systematic reviews, each paper highlights a different facet of how clinicians and researchers continue to address the inherent challenges of working in a small but complex field.

## 2. An Overview of Published Articles

This Special Issue encompassed 7 important original articles:

**1. Cardiac Manifestations and Persistent Myocardial Dysfunction in Multisystem Inflammatory Syndrome in Children: Insights from Conventional and Strain Echocardiography**—*Carmen-Corina Șuteu* et al.

This study explores the cardiac involvement in MIS-C using both conventional and strain echocardiography, revealing subtle yet persistent myocardial abnormalities. It exemplifies the complexity of pediatric cardiovascular disease, which often spans multiple age groups and requires sophisticated imaging tools to uncover hidden dysfunction—demonstrating how advanced diagnostics can bridge the gap between rarity and clinical relevance.

**2. Revisiting Hepatic Fibrosis Risk in Congenital Heart Disease: Insights from Non-Invasive Markers and Echocardiography**—*Fusako Yamazaki* et al.

This single-center study of 142 patients with CHD (i.e., Ventricular septal defects, Tetralogy of Fallot, Fontan) evaluates the burden of hepatic fibrosis using non-invasive biomarkers and echocardiography. Fibrosis was most pronounced in Fontan patients, but postoperative tetralogy of Fallot patients also showed a meaningful risk. aminotransferase-to-platelet ratio index and gamma-GT emerged as practical discriminators, with γ-GT ≥ 53 U/L indicating higher risk. Echocardiographic features, particularly reduced inferior vena cava mobility and respiratory variability, correlated with fibrosis severity. The study highlights the importance of structured hepatic surveillance to enable earlier detection and improve long-term outcomes in CHD survivors.

**3. Features of Clinical Manifestations and Heart Rate Variability in Children with Malignant Vasovagal Syncope**—*Wenrui Xu* et al.

This case–control study compared 10 children with malignant vasovagal syncope (VSS) to 40 age- and sex-matched non-asystolic VVS controls. Malignant cases showed earlier symptom onset and a markedly higher prevalence of central triggers and convulsive or incontinence episodes. Heart rate variability analysis revealed significantly elevated very low frequency, low frequency, and high frequency components, indicating pronounced autonomic imbalance with parasympathetic dominance. Central triggers and convulsive/incontinence manifestations emerged as independent risk factors for malignant VVS. The findings emphasize the need for early recognition of high-risk VVS phenotypes in pediatric patients.

**4. Analysis of Factors Relevant to the Severity of Symptoms in Children and Adolescents with Postural Orthostatic Tachycardia Syndrome**—*Yali Cao* et al.

This retrospective study examined 296 children and adolescents with newly diagnosed postural orthostatic tachycardia syndrome (POTS) to identify predictors of symptom severity. Symptom scores were correlated with clinical and ECG variables, revealing that corrected QT interval dispersion (QTcd) was the only independent factor associated with severity. QTcd showed excellent discriminatory power (AUC 0.986), with a cutoff of 45 ms providing high sensitivity and specificity for identifying severe cases. The study highlights QTcd as a simple, non-invasive marker that may help stratify risk and guide the management of pediatric POTS.

**5. Nomogram Based on Heart Rate Variability for Predicting the Therapeutic Efficacy of Orthostatic Training in Paediatric Vasovagal Syncope**—*Xiaojuan Du* et al.

By developing an HRV-based nomogram to predict treatment response in children with vasovagal syncope, this study shows how data-driven innovation can personalize therapy even in conditions with small and heterogeneous populations. It also reflects the challenge of working across different pediatric developmental stages, where physiological variability can profoundly influence therapeutic outcomes.

**6. Management of Paediatric Cardiac Arrest due to Shockable Rhythm—A Simulation-Based Study at Children’s Hospitals in a German Federal State**—*Nadine Mand* et al.

This simulation-based study investigates the response to cardiac arrest with shockable rhythms in pediatric hospitals, underscoring the importance of collaboration, training, and preparedness in rare but life-threatening emergencies. It also highlights the ethical and operational complexity of designing interventions specifically for children rather than adapting adult protocols.

**7. Relation between neutrophil count and left ventricular ejection 2 fraction following acute myocarditis in adolescents: a preliminary study**—*Barbara Rabiega* et al.

In this retrospective trial, the authors further reinforce the concept that even very simple inflammatory biomarkers, readily obtainable from routine blood tests such as a standard complete blood count, may carry relevant prognostic information in acute myocarditis. In adolescents with preserved or mildly reduced systolic function, the authors demonstrate an association between acute-phase neutrophil count and subsequent left ventricular functional recovery, supporting the pathophysiological and clinical relevance of innate immune activation. These findings are consistent with and complementary to previous large multicentre studies showing the prognostic value of the neutrophil-to-lymphocyte ratio across the left ventricular ejection fraction spectrum, including patients traditionally considered at lower risk [6,7]. Together, these data highlight how low-cost, widely available inflammatory indices may meaningfully contribute to early risk stratification and follow-up strategies in myocarditis.

Moreover, the Special Issue also includes 4 useful and comprehensive reviews:

**8. Pediatric Cardio-Oncology: Screening, Risk Stratification, and Prevention of Cardiotoxicity Associated with Anthracyclines**—*Xiaomeng Liu*

This comprehensive review of cardio-oncology in children emphasizes the need for early identification and prevention of anthracycline-induced cardiotoxicity. It reminds us that pediatric cardiac conditions differ not only in presentation but also in their lifelong implications—requiring specialized follow-up, multicenter collaboration, and shared data to improve long-term outcomes.

**9. Echocardiographic Assessment of Biventricular Mechanics of Fetuses and Infants of Gestational Diabetic Mothers: A Systematic Review and Meta-Analysis**—*Andrea Sonaglioni* et al.

This systematic review analyses ventricular function in fetuses and infants of diabetic mothers, offering crucial insights into the earliest stages of cardiovascular adaptation. It illustrates how pediatric cardiology must consider an entire spectrum of life—from the prenatal to the adolescent period—and how multicenter data integration can reveal subtle developmental mechanisms often hidden in small single-center studies.

**10. Towards a Multidisciplinary Approach of ECG Screening in Children and Adolescents: A Scoping Review (2005–2025)**—*Giovanna Zimatore* et al.

This scoping review charts twenty years of literature on electrocardiogram screening in young people, revealing how surprisingly thin the evidence remains for those under 16. From more than two decades of published work, only 14 eligible studies emerged, underscoring the fragmented nature of current knowledge. Procedures and protocols vary widely, and objective criteria to distinguish growth-related ECG changes from early signs of cardiac disease are still insufficient. The authors call for a coordinated, multidisciplinary framework that can refine interpretation, harmonize screening strategies, and improve early detection across pediatric and adolescent populations.

**11. Cardiopulmonary Exercise Testing in Congenital Heart Disease: A Never-Ending Story from Paediatrics to Adult Life**—*Giulia Guglielmi*, *Giorgia Rocchetti* et al.

This review follows the lifelong arc of patients with CHD through the lens of cardiopulmonary exercise testing (CPET), highlighting its growing role as a dynamic complement to resting evaluations. The authors examine how key parameters such as peak oxygen consumption, ventilatory efficiency, and heart rate responses inform prognosis, guide interventions, and shape individualized exercise recommendations across diverse CHD anatomies. While CPET consistently uncovers functional limitation and correlates with outcomes, certain metrics show variable reliability in complex physiologies, including cyanotic forms. In children, interpretation requires attention to growth-related changes, yet its value in assessing quality of life and therapeutic response is steadily expanding. Overall, the review positions CPET as an essential, non-invasive tool across the entire lifespan of CHD care, enabling more precise risk stratification and supporting active, safer living.

## 3. Conclusions

As this *Special Issue* demonstrates, pediatric cardiology remains both a demanding and inspiring frontier of medical science. Each contribution in this collection exemplifies how creativity, collaboration, and technological innovation can help overcome the intrinsic challenges of small populations, developmental diversity, and ethical limitations. From fetal imaging to adolescent care, from genetic discovery to clinical simulation, the studies presented here reaffirm that progress in pediatric cardiology depends not only on scientific excellence but also on shared vision and teamwork across disciplines and borders.

We extend our sincere gratitude to all authors, reviewers, and collaborators who contributed their expertise and enthusiasm to this issue. Their collective effort advances our understanding of the pediatric heart—one small step at a time, yet with a profound impact on the lives of children and families worldwide.

## Data Availability

Not applicable.
